# When Recurrent Pancreatitis Is Not Pancreatitis: Cyclic Vomiting Syndrome Masquerading as Acute Pancreatitis in a Young Adult

**DOI:** 10.7759/cureus.102682

**Published:** 2026-01-31

**Authors:** Niyas Khalid Ottu Para, Sumayya Kalakappara, Muhammed Thalha Manalodi Abubacker

**Affiliations:** 1 Internal Medicine, Burjeel Hospital, Abu Dhabi, ARE; 2 General Medicine, Aster Clinic, Remraam, Dubai, ARE; 3 General Medicine, Government Medical College, Manjeri, Manjeri, IND

**Keywords:** abdominal migraine, cyclical vomiting syndrome, gut-brain axis, hyperlipasemia, recurrent pancreatitis

## Abstract

Recurrent acute pancreatitis in young adults usually triggers an extensive evaluation for structural, genetic, metabolic, autoimmune, or toxic etiologies. However, pancreatic enzyme elevation and radiologic changes may occasionally arise from extra-pancreatic mechanisms, leading to diagnostic anchoring. We report a 20-year-old male who experienced four discrete episodes over a year, initially diagnosed as acute pancreatitis based on abdominal pain, vomiting, marked lipase elevation, and computed tomography findings. Each episode followed a highly stereotyped pattern beginning with prodromal uneasiness, progressing to repetitive vomiting, severe abdominal pain, diarrhea, and a post-episode phase of constipation with complete inter-episodic recovery. Subsequent attacks reproduced identical symptoms despite normal to mildly elevated pancreatic enzymes and imaging. The patient also reported recurrent sinus congestion, headaches, and muscle cramps, with a strong family history of migraine. An extensive evaluation, including heavy metal screening, autoimmune and immunologic testing, IgG4 levels, celiac serology, calcium and parathyroid hormone levels, ceruloplasmin, iron indices, cystic fibrosis transmembrane conductance regulator (CFTR) gene analysis, and porphyria testing, was unrevealing. Upper gastrointestinal endoscopy performed shortly after one attack demonstrated proximal gastric congestion without erosions or ulceration. Careful reconstruction of the clinical chronology revealed a classic four-phase cyclic pattern consistent with cyclic vomiting syndrome (CVS) within the abdominal migraine spectrum. Initiation of migraine-oriented prophylaxis with topiramate resulted in a marked reduction in attack frequency and severity. This case highlights how CVS can masquerade as recurrent pancreatitis and underscores the importance of recognizing neuro-visceral patterns in patients with recurrent vomiting and abdominal pain, even in the presence of transient pancreatic enzyme elevation.

## Introduction

Recurrent abdominal pain and vomiting in young adults represent a common and challenging clinical presentation. When such episodes are accompanied by elevated pancreatic enzymes or radiologic findings suggestive of pancreatitis, clinicians often anchor on a diagnosis of acute or recurrent pancreatitis [1,2]. However, pancreatic enzyme elevations, particularly modest or transient increases, are not specific for pancreatic injury and may arise from extra-pancreatic mechanisms, including vomiting, salivary enzyme release, systemic illness, or autonomic stress [3,4].

Cyclic vomiting syndrome (CVS) is a disorder of gut-brain interaction characterized by recurrent, stereotyped episodes of severe nausea and vomiting with complete inter-episodic recovery [5]. Although historically regarded as a pediatric condition, CVS is increasingly recognized in adults, where it remains underdiagnosed and frequently mislabeled as functional vomiting, gastroparesis, or unexplained abdominal pain [6,7]. CVS and abdominal migraine are now understood to lie within the migraine spectrum, sharing overlapping pathophysiologic mechanisms related to autonomic dysregulation, altered hypothalamic signaling, and neuronal hyperexcitability [8,9].

Adult practice guidelines from the American Neurogastroenterology and Motility Society (ANMS) and Cyclic Vomiting Syndrome Association (CVSA) emphasize the importance of recognizing stereotyped episodic patterns, symptom-free intervals, and comorbid migraine features, while excluding structural or metabolic disease before establishing the diagnosis [10]. Failure to recognize this pattern may result in repeated hospitalizations, unnecessary investigations, and delayed initiation of effective migraine-oriented prophylactic therapy.

We report a case in which early episodes fulfilled conventional criteria for acute pancreatitis, including biochemical and radiologic features, creating a compelling yet misleading diagnostic narrative. Only later, through careful reconstruction of the episodic pattern and exclusion of organic disease, was the underlying diagnosis of CVS within the abdominal migraine spectrum established.

The case underscores how a neuro-visceral syndrome can masquerade as recurrent pancreatitis and why clinicians must maintain a high index of suspicion for CVS in such scenarios.

## Case presentation

A 20-year-old previously healthy male was admitted with sudden-onset, severe epigastric abdominal pain following several hours of relentless vomiting. The episode began in a manner he would later recognize as characteristic, with a vague but compelling sense of internal uneasiness, described as something “not quite right inside,” followed by progressive nausea, pallor, and restlessness. Within hours, this evolved into repetitive, non-bilious vomiting, leaving him exhausted and diaphoretic.

As the vomiting subsided, the abdominal pain intensified and was described as deep and gripping across the upper abdomen. This phase was subsequently followed by loose stools. Over the next two to three days, as the pain gradually settled, he experienced marked fatigue and constipation, often passing no stool for 48-72 hours, after which he returned to complete baseline normality.

During this first presentation, pancreatic enzymes were elevated, with marked elevation of lipase and amylase rising to less than three times the upper limit of normal. Computed tomography of the abdomen was reported as consistent with acute pancreatitis, however, there was no evidence of necrosis, peripancreatic collections, or pancreatic ductal abnormalities. He was managed conservatively as acute pancreatitis and showed clinical improvement.

Over the subsequent year, the patient experienced three further hospital admissions with strikingly similar clinical episodes. Each episode followed the same sequence of prodromal uneasiness, intense vomiting, severe abdominal pain, diarrhea, and recovery accompanied by a transient period of constipation. During one of these early recurrent episodes, pancreatic enzymes were again modestly elevated, reinforcing the working diagnosis of recurrent acute pancreatitis.

Evolution of the clinical picture

The diagnostic narrative began to unravel when later episodes reproduced the same dramatic symptom pattern but occurred in the absence of pancreatic enzyme elevation and with entirely normal cross-sectional imaging. During one such admission, serum amylase and lipase were within normal limits, inflammatory markers were low, and repeated ultrasound of the abdomen was unremarkable.

An upper gastrointestinal endoscopy performed shortly after one of the later attacks demonstrated marked congestion of the proximal stomach without erosions or ulceration. Between episodes, the patient was entirely asymptomatic. He denied alcohol consumption, smoking, or recreational drug use. There was no history of gallstone disease, hypertriglyceridemia, or abdominal trauma.He has history of recurrent sinusitis and had undergone functional endoscopic sinus surgery (FESS). During attacks, he also reported recurrent nasal congestion with sinus pressure, episodic headaches, muscle cramps with generalized weakness, and pronounced fatigue during the recovery phase.

Family history was notable for maternal asthma and chronic sinus disease requiring functional endoscopic sinus surgery, as well as multiple relatives affected by migraine. There was also a family history of hypothyroidism, autoimmune in etiology. There was no family history of pancreatitis, inflammatory bowel disease, or celiac disease.

Investigations

Given the apparent diagnosis of recurrent pancreatitis in a young adult and the subsequent discordance between clinical symptoms and pancreatic enzyme levels and imaging findings, an exhaustive diagnostic evaluation was undertaken.

Laboratory and imaging workup

Initial laboratory investigations demonstrated leucocytosis with neutrophilia during the first episode, a pattern that was reproduced during subsequent attacks. Pancreatic enzymes showed marked elevation during the index episode, with progressively lower elevations during subsequent attacks, eventually normalizing during later episodes. Serum amylase was elevated initially and remained normal thereafter.

Serum total protein and albumin showed mild elevations, while lipid profile demonstrated mild hypercholesterolemia with elevated low-density lipoprotein levels and normal triglycerides. Renal function, electrolyte levels, inflammatory markers, and the remainder of the complete blood count were within normal limits.

Extensive toxic and metabolic evaluation, including screening for heavy metals, iron overload, and disorders of copper metabolism, was unremarkable. Autoimmune and immunologic testing, including IgG4 subclass levels, antinuclear antibodies, extractable nuclear antigen profile, smooth muscle antibodies, liver-kidney microsomal antibodies, complement levels, and total IgE was within reference ranges. Thyroid function testing revealed mildly elevated thyroid-stimulating hormone with no biochemical evidence of hypothyroidism.

Liver function tests and gamma-glutamyl transferase were normal. Serum calcium and parathyroid hormone levels were within reference ranges, and faecal pancreatic elastase was normal, arguing against exocrine pancreatic insufficiency.

Celiac disease screening, including tissue transglutaminase IgA and total IgA levels, was negative. Evaluation for acute hepatic porphyria, including urinary porphobilinogen testing performed shortly after a symptomatic episode, was normal.

**Figure 1:**
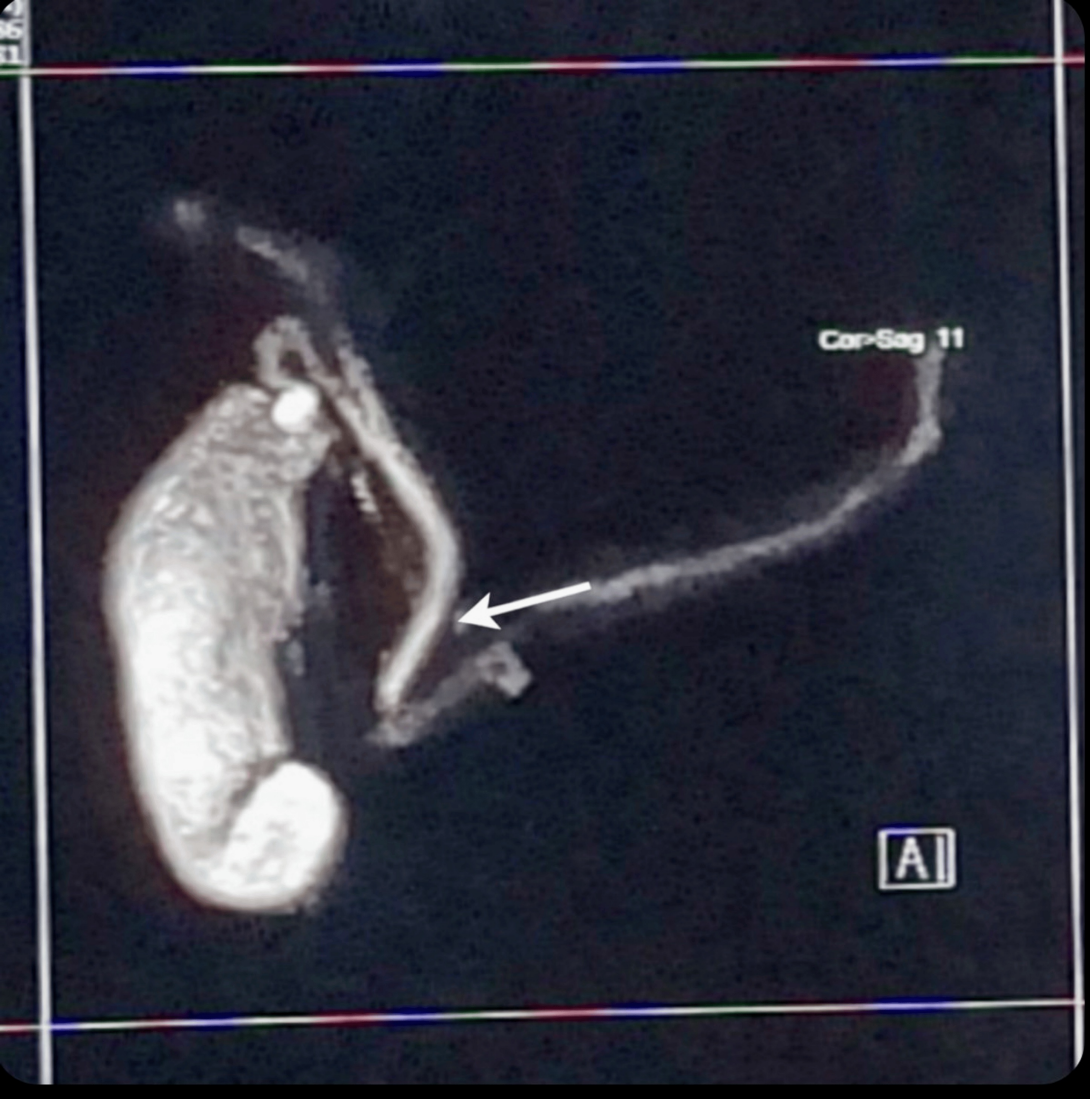
Magnetic resonance cholangiopancreatography (MRCP) MRCP image demonstrating normal pancreatico-biliary anatomy. The arrow highlights the normal caliber and smooth course of the pancreatic duct, without evidence of ductal dilatation, obstruction, or calculi.

**Figure 2:**
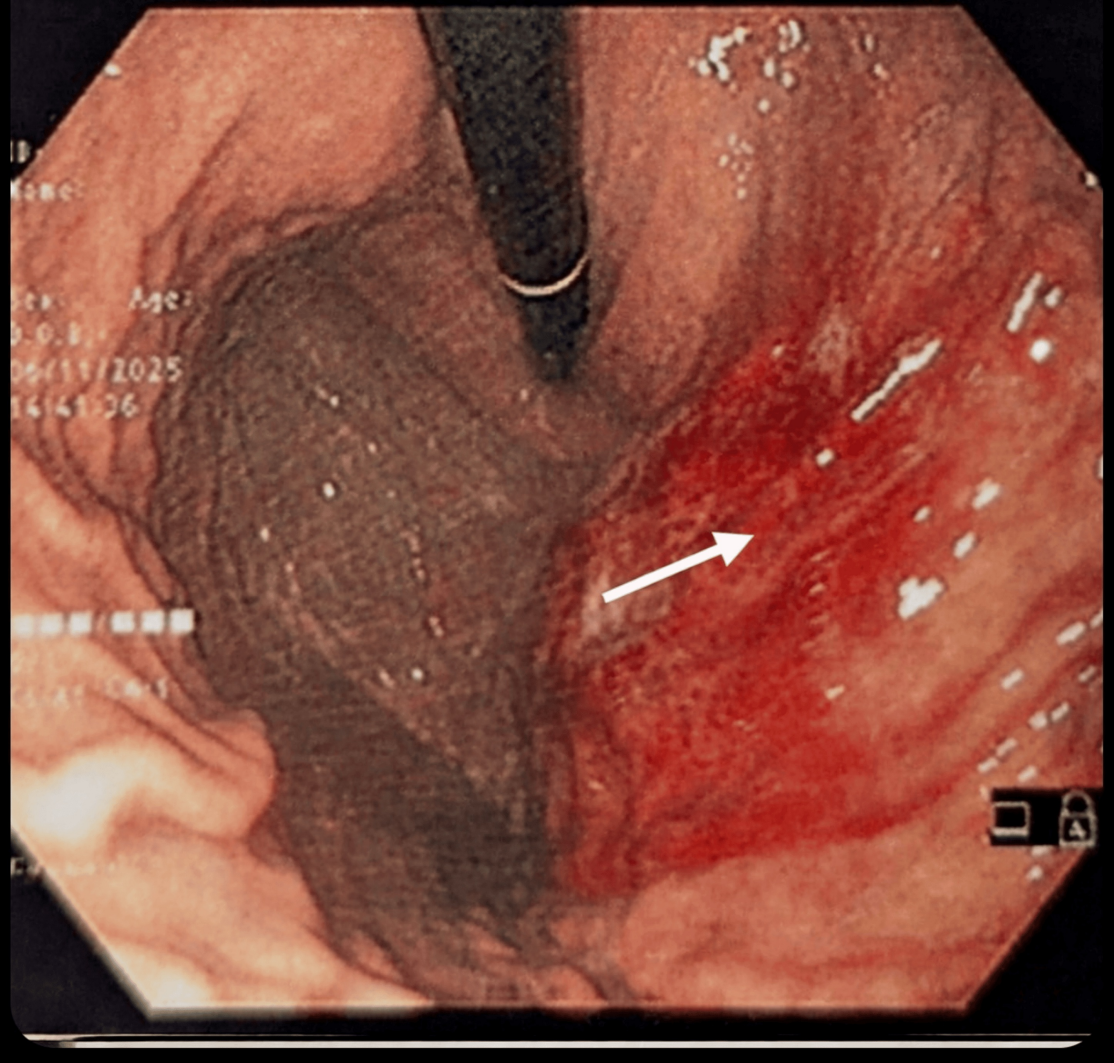
Upper gastrointestinal endoscopy Endoscopic image obtained shortly after a symptomatic episode demonstrating marked mucosal congestion and erythema involving the proximal gastric body along the greater curvature (arrow), without erosions or ulceration.

Abdominal ultrasonography performed after the first episode demonstrated no sonographic abnormalities, with visualization of the pancreatic head and proximal body. Magnetic resonance cholangiopancreatography performed after the second episode revealed normal pancreaticobiliary anatomy without ductal dilatation or calculi (Figure 1). Upper gastrointestinal endoscopy performed after the fourth episode demonstrated marked congestion of the proximal gastric body along the greater curvature (Figure 2). Original computed tomography images were not available for review, as these investigations were performed during earlier hospitalizations at an external institution.

 Table 1 shows the summary of investigations performed during the evaluation of recurrent pancreatitis-like episodes.

**Table 1:** Relevant laboratory values and investigations

Category	Test / investigation	Result	Reference range / interpretation
Pancreatic Enzymes	Serum lipase (peak)	3367 U/L	<60 U/L
Pancreatic Enzymes	Serum lipase (subsequent episodes)	721 → 305 → 110 U/L	Progressive normalization
Pancreatic Enzymes	Serum amylase	Mildly elevated initially, later normal	<100 U/L
Inflammatory Markers	CRP / ESR	Normal	No systemic inflammation
Metabolic	Serum calcium	Normal	8.5–10.5 mg/dL
Metabolic	Parathyroid hormone (PTH)	Normal	15–65 pg/mL
Iron / Copper Metabolism	Ferritin / transferrin saturation	Normal	Iron overload excluded
Iron / Copper Metabolism	Ceruloplasmin	Normal	Wilson disease unlikely
Autoimmune / Immune	IgG4 subclass	Normal	<135 mg/dL
Autoimmune / Immune	ANA, anti-dsDNA, and anti-CCP	Negative	Systemic autoimmune disease excluded
Autoimmune / Immune	SMA / LKM antibodies	Negative	Autoimmune hepatitis excluded
Autoimmune / Immune	Complement C3 / C4	Normal	No immune consumption
Autoimmune / Immune	Total IgE	Normal	No allergic or atopic driver
Celiac / Inflammatory	TTG IgA / total IgA	Negative / Normal	Celiac disease excluded
Celiac / Inflammatory	Stool calprotectin	Normal	<50 µg/g; IBD unlikely
Porphyria Screening	Urine PBG and ALA	Negative	Acute hepatic porphyria excluded
Genetic	CFTR full gene sequencing	Negative	CFTR-related disorder unlikely
Toxicology	Heavy metals (Pb, Hg, As, and Cd)	Negative	Toxic etiology excluded
Imaging	MRCP	Normal pancreatic ductal anatomy	Structural causes excluded
Endoscopy	Upper GI endoscopy	Congested proximal stomach	Reactive vascular congestion, no inflammation

Diagnostic reasoning and differential diagnosis

Recurrent Idiopathic Pancreatitis

The initial working diagnosis of recurrent acute pancreatitis was supported by the presence of epigastric abdominal pain, vomiting, transient pancreatic enzyme elevation during early episodes, and radiologic interpretation suggestive of pancreatitis. However, this explanation became increasingly untenable when subsequent attacks occurred with normal pancreatic enzymes and imaging, in the absence of gallstones, alcohol exposure, hypercalcemia, or hypertriglyceridemia. Genetic causes of pancreatitis were considered; however, CFTR gene testing was negative, and other mutations were felt to have a low pre-test probability.

It is well recognized in the literature that modest elevations in amylase and lipase are not specific to pancreatitis and may occur in non-pancreatic conditions, including vomiting, salivary pathology, and systemic illness.

CFTR-Related Disorder

Given the combination of sinus symptoms, possible pancreatic involvement, and young age, a CFTR-related disorder was considered as a unifying diagnosis. Adult presentations of CFTR-related disease may include recurrent pancreatitis and sino-nasal disease in the absence of overt pulmonary manifestations. However, comprehensive CFTR gene sequencing was negative, making this diagnosis unlikely.

Porphyria and Metabolic Channelopathies

The episodic nature of symptoms combined with abdominal pain and neuromuscular complaints prompted evaluation for acute hepatic porphyria and metabolic channelopathies. Normal urinary porphobilinogen testing performed during an attack effectively excluded acute hepatic porphyria. Other metabolic and mitochondrial disorders were considered but lacked supportive biochemical or clinical features.

Abdominal Migraine and CVS

The diagnostic turning point occurred when the chronology of symptoms was reconstructed in detail. Each episode began with a prodromal phase characterized by vague malaise and anticipatory distress, progressed to repetitive and often severe vomiting, evolved into intense abdominal pain with associated diarrhea, and resolved over several days with post-episode constipation and profound fatigue. Complete wellness was observed between episodes.

This highly stereotyped four-phase pattern closely mirrors descriptions of CVS and abdominal migraine in the literature. The strong family history of migraine further supported a migraine-spectrum episodic disorder. Adult CVS case series describe similar diagnostic delays, extensive negative investigations, and frequent mislabeling as functional gastrointestinal disorders or atypical pancreatitis. Current guidelines emphasize that recurrent pancreatitis must be excluded before a diagnosis of CVS is made.

Based on the characteristic episodic pattern, complete inter-episodic recovery, migraine diathesis, and exhaustive exclusion of organic disease, a diagnosis of CVS within the abdominal migraine spectrum was established.

Following the establishment of a diagnosis of CVS within the abdominal migraine spectrum, the patient was initiated on prophylactic therapy with topiramate. Since initiation of treatment, he has remained symptom-free, with no recurrence of vomiting, abdominal pain, or pancreatitis-like episodes over the follow-up period to date. This clinical response further supports the diagnosis of a migraine-spectrum neuro-visceral disorder.

## Discussion

Diagnostic delay in CVS

CVS and abdominal migraine are now recognized as episodic disorders within the migraine spectrum, characterized by recurrent attacks of nausea, vomiting, and abdominal pain lasting hours to days, with complete remission between episodes [5,8]. Although historically regarded as pediatric conditions, adult presentations of CVS are increasingly reported. Adult case series consistently describe prolonged diagnostic delays, repeated hospitalizations, and a high burden of unnecessary investigations before the correct diagnosis is established [6,7].

Fleisher and colleagues described a cohort of 41 adults with CVS, highlighting highly stereotyped episodes, complete symptom-free intervals, and frequent comorbid migraine and anxiety disorders [6]. Subsequent reports by Tang et al. and Duckett et al. similarly demonstrated that recurrent vomiting and abdominal pain in adults are often misattributed to functional gastrointestinal disorders or unexplained abdominal pain syndromes for years before CVS is recognized [7,11]. This diagnostic trajectory closely mirrors the experience of the patient described in this case.

The ANMS and CVSA guidelines provide structured diagnostic criteria for adult CVS, emphasizing the presence of stereotyped acute episodes, at least three discrete events, complete inter-episodic wellness, and exclusion of structural or metabolic disease [10]. Our patient fulfilled each of these criteria, and the exhaustive diagnostic evaluation further supported a diagnosis within the spectrum of disorders of gut-brain interaction.

Pathophysiologic considerations 

CVS is conceptualized as a disorder of gut-brain interaction in which autonomic instability, hypothalamic dysregulation, and migraine-related neural mechanisms converge on emetic and visceral pain pathways [8,9]. Abdominal migraine and CVS share overlapping pathophysiologic substrates, with migraine circuitry manifesting through visceral rather than cephalic symptoms.

Levinthal and colleagues have proposed that adult CVS represents a failure of allostatic regulation, whereby physiological or psychological stressors-such as sleep deprivation, fasting, infection, or emotional stress-push a vulnerable system beyond a threshold, resulting in episodic symptomatic crises [12]. In the present case, attacks were frequently preceded by nonspecific prodromal uneasiness and autonomic symptoms, a pattern that aligns closely with this threshold-based model and supports a neuro-visceral rather than structural gastrointestinal disorder.

Why the presentation mimicked recurrent pancreatitis

The initial diagnosis of recurrent acute pancreatitis was driven by the convergence of severe epigastric abdominal pain, repetitive vomiting, and transient elevation of pancreatic enzymes accompanied by radiologic interpretations suggestive of pancreatitis during early episodes. However, substantial literature demonstrates that elevations in serum amylase and lipase are not specific for pancreatic injury and may occur in a wide range of non-pancreatic conditions [3,4].

Vomiting alone has been shown to elevate serum amylase, often through increased salivary isoamylase, as documented in hyperemesis gravidarum and eating disorders [13]. Systemic illness and autonomic stress may also contribute to transient hyperlipasemia in the absence of structural pancreatic disease. In this patient, intense vomiting and autonomic activation likely produced a transient biochemical and radiologic signal that was interpreted as pancreatitis, leading to early diagnostic anchoring.

Once the pancreatitis label was applied, subsequent clinically identical episodes were interpreted through this framework, even when pancreatic enzymes and imaging were normal. This illustrates how early biochemical and radiologic findings can disproportionately influence diagnostic reasoning. Notably, recurrent pancreatitis is explicitly listed among the differential diagnoses that must be excluded before diagnosing CVS, underscoring the significant clinical overlap between these entities [14].

Proximal gastric congestion and autonomic dysregulation

The finding of marked proximal gastric congestion on endoscopy shortly after an attack initially raised concern for primary gastric pathology or intermittent structural abnormalities. However, in the context of CVS, this finding is physiologically plausible and consistent with autonomic vasomotor dysregulation.

Reactive mucosal hyperemia following repeated vomiting, combined with autonomic vascular instability during the recovery phase, provides a unifying explanation for this endoscopic appearance. The absence of erosions or ulceration on endoscopy supports a functional rather than inflammatory etiology. Although systematic data on post-episode endoscopic findings in CVS are limited, this pattern aligns with current models of autonomic involvement in gut-brain interaction disorders and should be interpreted cautiously to avoid diagnostic misdirection [8].

Genetic susceptibility and familial migraine diathesis

Although no pathogenic variants were identified on targeted genetic testing in this patient, the strong family history of migraine provides an important contextual framework for understanding his clinical presentation. CVS and abdominal migraine are increasingly regarded as phenotypic expressions of inherited migraine susceptibility, in which genetic vulnerability confers a predisposition to episodic neuro-visceral dysregulation rather than a single-organ pathology.

Migraine is a highly heritable disorder, with heritability estimates ranging from 40% to 60% [15]. Multiple genetic loci related to neuronal excitability, ion channel regulation, mitochondrial energy metabolism, and neurotransmitter signaling have been implicated [16]. Rather than a monogenic disorder, migraine appears to arise from polygenic susceptibility interacting with environmental and physiological triggers.

Emerging evidence suggests that CVS shares mechanistic overlap with migraine-related channelopathies and mitochondrial dysfunction, particularly involving calcium and sodium channel regulation and impaired cellular energy homeostasis. These mechanisms may explain why individuals with a strong familial migraine background develop visceral manifestations such as cyclic vomiting, abdominal pain, and autonomic symptoms rather than classic cephalic headache. The presence of episodic headaches, prodromal autonomic symptoms, and complete inter-episodic recovery in our patient further supports this migraine-spectrum model.

The family history of autoimmune hypothyroidism may also be relevant, as immune-mediated and inflammatory pathways have been proposed as modulators of migraine susceptibility and autonomic tone. While speculative, this observation underscores the multifactorial nature of neuro-visceral disorders and the interplay between genetic, immunologic, and environmental factors.

Taken together, the patient’s familial migraine background likely lowered the neurobiological threshold for episodic dysregulation of the gut-brain axis. This genetic predisposition provides a unifying explanation for the stereotyped cyclic pattern, autonomic features, and robust response to migraine-oriented prophylaxis with topiramate.

Treatment and clinical outcome

Current guidelines recommend tricyclic antidepressants, particularly amitriptyline, as first-line prophylactic therapy for moderate-to-severe adult CVS, with alternatives including topiramate and aprepitant [10]. Topiramate exerts its therapeutic effect through modulation of voltage-gated ion channels, enhancement of inhibitory gamma-aminobutyric acid activity, and attenuation of glutamatergic neurotransmission-mechanisms that align closely with the neuroexcitability framework of CVS.

Retrospective adult series and pediatric studies have demonstrated that topiramate can significantly reduce the frequency and severity of CVS episodes, including in patients with atypical or red-flag features [17,18]. In this case, initiation of low-dose topiramate with gradual titration resulted in a marked reduction in attack frequency and intensity, further reinforcing the diagnostic conclusion.

Clinical significance and educational implications

This case is notable because, unlike many reported adult CVS cases misdiagnosed as functional vomiting or gastroenteritis, the early episodes fulfilled conventional criteria for acute pancreatitis, including biochemical and radiologic features. This created a compelling but ultimately misleading diagnostic narrative.

The case illustrates how vomiting-induced and autonomically mediated enzyme elevations can generate a “functional pancreatitis” phenotype in the absence of structural disease, highlighting the importance of cautious interpretation of modest or transient pancreatic enzyme elevations. The extensive negative diagnostic workup demonstrates how disorders of gut-brain interaction can convincingly masquerade as complex systemic disease.

From an educational perspective, this case underscores the role of cognitive biases such as anchoring and availability in clinical reasoning and emphasizes the value of pattern recognition in episodic syndromes. It also raises the possibility that a subset of young adults labeled with recurrent idiopathic pancreatitis may, in fact, have underlying CVS or abdominal migraine, particularly when structural and genetic causes are excluded.

## Conclusions

This case highlights CVS as a neuro-visceral impostor capable of masquerading as recurrent acute pancreatitis in a young adult. Early episodes accompanied by modest pancreatic enzyme elevation and non-specific computed tomography findings anchored the diagnosis to pancreatic pathology, delaying recognition of an underlying cyclic, migraine-spectrum disorder of gut-brain interaction.

In patients presenting with recurrent vomiting and abdominal pain, careful attention should be paid to stereotyped symptom cycles and complete inter-episodic recovery. Diagnostic reconsideration is warranted when pancreatic enzymes and imaging normalize despite persistence of an identical clinical pattern. Incorporation of family history of migraine and atopic disease into diagnostic reasoning is essential, as modest hyperamylasemia or hyperlipasemia may reflect vomiting or autonomic stress rather than structural pancreatic injury. Early recognition of CVS can prevent unnecessary investigations and procedures, reduce healthcare utilization, and facilitate targeted management with migraine-oriented prophylactic therapy such as amitriptyline or topiramate, alongside lifestyle modification and trigger avoidance.
